# Engagement Among Diverse Patient Backgrounds in a Remote Symptom Monitoring Program

**DOI:** 10.1200/OP.24.00066

**Published:** 2024-06-25

**Authors:** Gabrielle B. Rocque, Nicole E. Caston, Keyonsis Hildreth, Luqin Deng, Nicole L. Henderson, Courtney P. Williams, Andres Azuero, Bradford E. Jackson, Jeffrey A. Franks, Chelsea McGowan, Chao-Hui Sylvia Huang, D'Ambra Dent, Stacey Ingram, J. Nicholas Odom, Noon Eltoum, Bryan Weiner, Doris Howell, Angela M. Stover, Jennifer Young Pierce, Ethan Basch

**Affiliations:** ^1^ Department of Medicine, Division of Hematology and Oncology, University of Alabama at Birmingham, Birmingham, AL; ^2^ O'Neal Comprehensive Cancer Center, Birmingham, AL; ^3^ Department of Medicine, Division of Preventive Medicine, University of Alabama at Birmingham, Birmingham, AL; ^4^ School of Nursing, University of Alabama at Birmingham, Birmingham, AL; ^5^ Lineberger Comprehensive Cancer Center, University of North Carolina at Chapel Hill, Chapel Hill, NC; ^6^ University of South Alabama Mitchell Cancer Institute, Mobile, Alabama; ^7^ Department of Medicine, Division of Gerontology, Geriatrics, and Palliative Care, University of Alabama at Birmingham, Birmingham, AL; ^8^ Department of Radiology, University of Alabama at Birmingham, Birmingham, AL; ^9^ Department of Health Systems and Population Health, University of Washington, Seattle, WA; ^10^ Supportive Care, Princess Margaret Cancer Centre Research Institute, Toronto, ON, Canada; ^11^ Department of Health Policy and Management, University of North Carolina at Chapel Hill, Chapel Hill, NC

## Abstract

**PURPOSE:**

Previous randomized controlled trials have demonstrated benefit from remote symptom monitoring (RSM) with electronic patient-reported outcomes. However, the racial diversity of enrolled patients was low and did not reflect the real-world racial proportions for individuals with cancer.

**METHODS:**

This secondary, cross-sectional analysis evaluated engagement of patients with cancer in a RSM program. Patient-reported race was grouped as Black, Other, or White. Patient address was used to map patient residence to determine rurality using Rural-Urban Commuting Area Codes and neighborhood disadvantage using Area Deprivation Index. Key outcomes included (1) being approached for RSM enrollment, (2) declining enrollment, (3) adherence with RSM via continuous completion of symptom surveys, and (4) withdrawal from RSM participation. Risk ratios (RR) and 95% CI were estimated from modified Poisson models with robust SEs.

**RESULTS:**

Between May 2021 and May 2023, 883 patients were approached to participate, of which 56 (6%) declined RSM. Of those who enrolled in RSM, a total of 27% of patients were Black or African American and 67% were White. In adjusted models, all patient population subgroups of interest had similar likelihoods of being approached for RSM participation; however, Black or African American patients were more than 3× more likely to decline participation than White participants (RR, 3.09 [95% CI, 1.73 to 5.53]). Patients living in more disadvantaged neighborhoods were less likely to decline (RR, 0.49 [95% CI, 0.24 to 1.02]), but less likely to adhere to surveys (RR, 0.81 [95% CI, 0.68 to 0.97]). All patient populations had a similar likelihood of withdrawing.

**CONCLUSION:**

Black patients and individuals living in more disadvantaged neighborhoods are at risk for lower engagement in RSM. Further work is needed to identify and overcome barriers to equitable participation.

## Teaser

Black patients and individuals living in more disadvantaged neighborhoods are at risk for lower engagement in remote symptom monitoring

## INTRODUCTION

Capture of electronic patient-reported outcome (ePRO) data for remote symptom monitoring (RSM) provides opportunities for proactive symptom management to patients undergoing treatment for cancer. Randomized controlled trials have demonstrated benefits to RSM in terms of symptom reduction, quality of life, health care utilization, and survival.^1-4^ The recent PRO-TECT trial, which cluster randomized 52 community oncology practices across 21 states to RSM versus usual care, found that patients receiving RSM had improved quality of life, symptom control, and physical function.^3^ Furthermore, RSM was acceptable to patients as 90% of participating patients endorsed recommending the program to others.^5^ Because of the success across these and other studies, collection of ePROs is included as a requirement for oncology practices participating in Medicare's value-based care payment model, the Enhancing Oncology Model, spotlighting a need for implementation of RSM into standard of care at practices nationwide.^6^

**CONTEXT**

**Key Objective**
What are the rates of approach, enrollment, and participation of historically under-represented patient subgroups in a real-world, electronic patient-reported outcome-based remote symptom monitoring (RSM) program?
**Knowledge Generated**
In adjusted models, all patient population subgroups of interest had similar likelihoods of being approached for RSM participation; however, Black or African American patients were more than 3× more likely to decline participation than White participants (risk ratios [RR], 3.09 [95% CI, 1.73 to 5.53]). Patients living in more disadvantaged neighborhoods were less likely to decline (RR, 0.49 [95% CI, 0.24 to 1.02]), but less likely to adhere to surveys (RR, 0.81 [95% CI, 0.68 to 0.97]).
**Relevance**
Black patients and individuals living in more disadvantaged neighborhoods are at risk for lower engagement in RSM. Further work is needed to identify and overcome barriers to equitable participation.

Although previous trials have demonstrated efficacy of RSM, the racial diversity of patients has been limited and not representative of the US population of marginalized individuals with cancer. In a landmark study by Basch et al^7^ demonstrating clinical benefits for RSM, only 9% of study participants were Black or African American (henceforth referred to as Black) and few lived in rural areas. As cancer practices begin to implement RSM as the standard of care across more diverse patient populations,^8-10^ a better understanding of factors influencing engagement with RSM in diverse patient populations is needed to ensure equitable cancer care outcomes. This lack of understanding is problematic as inequitable implementation of previous screening programs has resulted in disparate outcomes. For example, overall breast and colorectal cancer mortality rates have significantly declined because of the initiation of screening and improved treatment options, yet mortality rates declined more significantly for White compared with Black patients.^11,12^ Furthermore, as health care interventions, such as RSM, are increasingly digitized, increased attention to digital health literacy and technology-related barriers in marginalized populations, such as Black patients, older patients, and those living in under-resourced and/or rural communities,^13,14^ is needed to ensure that disparities are not exacerbated.^15,16^

Assessing engagement and participation patterns for patients from historically marginalized populations is crucial for equitable RSM implementation. The University of Alabama at Birmingham (UAB) and University of South Alabama, both institutions located in a state with high poverty rates, 46% of residents living in rural areas and a population that is over one quarter Black,^17,18^ are implementing RSM across their entire cancer populations.^9^ This provides a unique opportunity to evaluate engagement in RSM of patients living in disadvantaged neighborhoods, individuals living in rural areas, and Black patients. This article assessed four engagement steps, which, if disparate, would require developing strategies for increased participation: (1) how eligible patients are identified by the clinical team and approached for RSM enrollment, (2) patient agreement for RSM participation, (3) patient adherence with RSM via continuous completion of symptom surveys, and (4) patient withdrawal of participation during the initial 2 years after program initiation.

## METHODS

### Study Design

This was a preplanned analysis from a hybrid, type 2 trial evaluating implementation and patient outcomes of RSM supported by lay navigators focused on patients living in disadvantaged neighborhoods, those residing in rural areas, and Black patients (ClinicalTrials.gov identifier: NCT04809740). The full trial design was previously described.^9^ This analysis focuses on implementation outcomes for patients in these populations during the initial program scale-up. This study was approved by the UAB Institutional Review Board (IRB-300007406).

### Program and Population

UAB O'Neal Comprehensive Cancer Center and the University of South Alabama Mitchell Cancer Institute (located in Mobile, AL) began formal standard-of-care RSM implementation in May 2021, rolling out in sequential disease-based teams over time. Year 1 target enrollment was 30% of eligible patients, with increase to 40% in year 2. This analysis includes data through May 2023. Patients who were new to participating institutions and receiving chemotherapy, immunotherapy, or targeted therapy (infusion or oral) were eligible for enrollment. Nonclinical navigators were responsible for enrollment. These individuals identify barriers to health care, address nonclinical concerns, triage clinical concerns to appropriate team members, and support care coordination.^19^ Navigators identified eligible patients using clinic and infusion appointment lists, confirmed with the clinical team via notes or dialogue. Postvisit electronic medical record (EMR) and billing information were used as secondary sources to identify eligible patients potentially missed during initial screening. Navigators enrolled the patient in person or on the phone in a web-based platform that was accessible on any smart device or computer. Smart phones with data capability were available at no cost for patients who lacked access to a device with internet access although only 11 individuals used this option from 2021 to 2024.

Enrolled patients completed weekly symptom assessment surveys for 6 months via an ePROs system (Carevive), third-party ePRO software integrated into the EMR of the participating cancer centers. Patients could continue RSM beyond 6 months on the basis of patient request or transition to a new therapy. If five consecutive surveys are not completed by the patient, RSM surveys are automatically discontinued, but the patient remains enrolled, so they can be reinitiated by the clinical team if and when desired. Symptom surveys with moderate or severe symptoms generated alerts within the EMR to clinic nurses, which prompted clinical management at the nurse's discretion using institutional protocols for symptom management. For this analysis, only White and Black patients were included because of small frequencies (<5%) for other races and ethnicities. Consent was waived as RSM was administered as part of standard of care.

### Data Sources

RSM data were abstracted from the patient-reported outcome platform. Patient clinical and treatment data were abstracted from the EMR and included date of birth, sex (female, male), insurance status (private, Medicaid, Medicare, none/other), cancer type (breast, GI, genitourinary, gynecologic, head and neck, hematologic [leukemia, lymphoma, myeloma], lung, melanoma, other), and cancer treatment (chemotherapies, immunotherapies, and targeted therapies). Missing EMR data were supplemented with data from billing records and data from the patient-reported outcome platform.

### Outcomes (dependent variables): RSM Engagement and Participation

This analysis included four key RSM engagement and participation outcomes:

1. Approached for RSM: Of eligible patients, the number of patients approached versus not approached by a lay navigator for enrollment was calculated.
2. Declined enrollment into RSM: Of approached patients, the number of patients who declined enrollment versus enrolled into RSM was quantified.
3. Adherence with RSM: Of enrolled patients, adherence was defined as the percentage of RSM surveys completed of those assigned (range, 0%-100%). The institutional adherence goal was set at 75% of survey completion for 6 months, on the basis of an anticipated decline when making the transition to a real-world setting from 90% survey completion in previous randomized controlled trials.^3^ Each patient had a mean adherence score and was considered either adherent or not according to the institutional cutoff of 75%.
4. Withdrew from RSM: Of enrolled patients, the number of patients who requested to withdraw at any time was captured. Patient adherence data from those who withdrew from RSM were included if their ePROs were captured before withdrawal.

### Exposures (independent variables): Patient Population Groups

This analysis included three key patient population group exposures:

1. Patient race: Race was self-reported as Black, Other (including American Indian or Alaska Native, Asian, Hispanic or Latino, Native Hawaiian or Pacific Island, Other), or White.
2. Patient residence: Rural-Urban Commuting Area Codes were used to assign patient residence as rural or urban using ZIP codes.^20^
3. Patient neighborhood disadvantage: The Area Deprivation Index (ADI) was used to determine patient neighborhood disadvantage using Census block data. ADI encompasses domains of education, income, employment, and housing quality at the neighborhood level. ADI scores range from 1 to 100, with higher scores representing greater neighborhood disadvantage. Neighborhoods with ADI scores from 86 to 100 are considered more disadvantaged.^21^

### Statistical Analysis

Descriptive statistics were calculated using frequencies and percentages for categorical variables and medians and IQR for continuous variables. Differences in characteristics for patients who were approached or not approached and who declined or enrolled were calculated using measures of effect size such as Cohen's *d* (ie, the standardized mean difference; small: 0.2, medium: 0.5, large: 0.8) for numerical characteristics or Cramer's *V* for cross-tabulations. *V* of 0.1 is considered a small effect, 0.3 is considered a medium effect, and 0.5 is considered a large effect when comparing across two categories; *V* of 0.1 is considered a small effect, 0.25 is considered a medium effect, and 0.4 is considered a large effect when comparing across more than two categories.^22^ Associations between patient population groups and RSM engagement and participation outcomes (ie, approached to participate, declined participation, adherence [according to 75% cutoff], and withdrawing from the program) were estimated using risk ratios (RR), predicted probabilities (presented as percentages out of 100), and 95% CI from modified Poisson models with robust SEs.^23-25^ Mean adherence by patient population groups was calculated as predicted probabilities using model-estimated means and 95% CIs from generalized linear models. All models were adjusted for patient race, residence, neighborhood disadvantage, age when approached for RSM participation, sex, insurance status, and enrollment institution. Multiple imputation was performed under a missing at random assumption to account for missing covariable data (range, 1%-5% missing; with a total of 13% of sample missing covariable data),^26^ resulting in 15 imputed data sets. Sensitivity analyses were performed using complete case analyses. Additional sensitivity analyses considering interactions between race and ADI were conducted using logistic regression models among the complete case data. Analyses were performed using SAS software, version 9.4 (SAS Institute, Cary, NC).

## RESULTS

### Patients Eligible for RSM

A total of 1,998 eligible patients were included in the analysis; 527 (26%) were Black, 318 (16%) were rural residents, and 477 (24%) lived in an ADI-defined highly disadvantaged neighborhood (Table 1; Appendix Table A1, online only). Patients eligible for RSM were most often diagnosed with breast (478 [24%]), hematologic (466 [23%]), or GI (361 [18%]) cancer, which was attributable to the sequence of RSM rollout and volume of patients across both institutions. Most eligible patients were treated at UAB (1,468 [73%]).

**TABLE 1.** Demographics and Clinical Characteristics of Patients Eligible for (n = 1,998), Approached for (n = 883), Not Approached for (n = 1,157), Declined to Enrollment in (n = 56), and Enrolled Into (n = 827) RSM

Characteristic	Eligible (n = 1,998)	Approached (n = 883)	Not Approached (n = 1,115)	Declined (n = 56)	Enrolled (n = 827)	Withdrawn (n = 133)
Age at approach or eligibility state (for those not approached), years, median (IQR)	62 (52-70)^a^ n = 1,955^a^	61 (51-69)	64 (53-72)^a^ n = 1,072^a^	69 (58-72)	60 (51-69)	60 (51-68)
Approached status, No. (%)						
Approached	883 (44)	883 (100)	0	56 (100)	827 (100)	133 (100)
Not approached	1,115 (56)	0	1,115 (100)	0	0	0
Declined status,^a^ No. (%)						
Declined	56 (3)	56 (6)	0	56 (100)	0	0
Enrolled	827 (42)	827 (94)	0	0	827 (100)	133 (100)
Enrollment status,^a^ No. (%)						
Enrolled	694 (35)	694 (79)	0	0	694 (84)	0
Withdrawn	133 (7)	133 (15)	0	0	133 (16)	133 (100)
Race, No. (%)						
Black or African American	527 (26)	250 (28)	277 (25)	23 (41)	227 (27)	39 (29)
Other	75 (4)	24 (3)	51 (5)	0	24 (3)	5 (4)
White	1,244 (62)	586 (66)	658 (59)	31 (55)	555 (67)	87 (65)
Unknown	152 (8)	23 (3)	129 (12)	2 (4)	21 (3)	2 (2)
RUCA,^b^ No. (%)						
Rural	318 (16)	132 (15)	186 (17)	10 (18)	122 (15)	16 (12)
Urban	1,541 (77)	704 (80)	837 (75)	43 (77)	661 (80)	107 (80)
Unknown	139 (7)	47 (5)	92 (8)	3 (5)	44 (5)	10 (8)
Area Deprivation Index, No. (%)						
Less disadvantaged	1,365 (68)	617 (70)	748 (67)	42 (75)	575 (70)	88 (66)
More disadvantaged	477 (24)	212 (24)	265 (24)	10 (18)	202 (24)	34 (26)
Unknown	156 (8)	54 (6)	102 (9)	4 (7)	50 (6)	11 (8)
Sex, No. (%)						
Female	1,172 (59)	603 (68)	569 (51)	27 (48)	576 (70)	99 (74)
Male	783 (39)	280 (32)	503 (45)	29 (52)	251 (30)	34 (26)
Unknown	43 (2)	0	43 (4)	0	0	0
Cancer type, No. (%)						
Breast	478 (24)	290 (33)	188 (17)	9 (16)	281 (34)	58 (44)
GI	361 (18)	158 (18)	203 (18)	24 (43)	134 (16)	22 (17)
Genitourinary	125 (6)	41 (5)	84 (8)	3 (5)	38 (5)	4 (3)
Gynecologic	190 (10)	121 (14)	69 (6)	2 (4)	119 (14)	12 (9)
Head and neck	73 (4)	24 (3)	49 (4)	2 (4)	22 (3)	5 (4)
Hematologic	466 (23)	120 (14)	346 (31)	13 (23)	107 (13)	13 (10)
Lung	137 (7)	79 (9)	58 (5)	2 (4)	77 (9)	14 (11)
Melanoma	73 (4)	23 (3)	50 (4)	0	23 (3)	1 (1)
Other	44 (2)	27 (3)	17 (2)	1 (2)	26 (3)	4 (3)
Unknown	51 (3)	0	51 (5)	0	0	0
Insurance status, No. (%)						
Medicaid	153 (8)	82 (9)	71 (6)	2 (4)	80 (10)	17 (13)
Medicare	781 (39)	335 (38)	446 (40)	32 (57)	303 (37)	49 (37)
None/other	251 (13)	82 (9)	169 (15)	6 (11)	76 (9)	7 (5)
Private	810 (41)	384 (43)	426 (38)	16 (29)	368 (44)	60 (45)
Unknown	3 (0)	0	3 (0)	0	0	0
Cancer center, No. (%)						
UAB	1,468 (73)	577 (65)	891 (80)	49 (88)	528 (64)	92 (69)
MCI	530 (27)	306 (35)	224 (20)	7 (13)	299 (36)	41 (31)
Compliance percentage, median (IQR)^c^					80 (44-100) n = 816^a^	83 (52-100)
Compliance percentage, dichotomized,^c^ No. (%)						
75% or greater					436 (53)	79 (59)
Below 75%					380 (47)	54 (41)

Abbreviations: MCI, Mitchell Cancer Institute; RSM, remote symptom monitoring; RUCA, Rural-Urban Commuting Area; UAB, University of Alabama at Birmingham.

^a^ Does not add up to 100% for all columns.

^b^ RUCA codes were rural areas which included 4.0, 4.2, 5.0, 5.2, 6.0, 6.1, 7.0, 7.2, 7.3, 7.4, 8.0, 8.2, 8.3, 8.4, 9.0, 9.1, 9.2, 10.0, 10.2, 10.3, 10.4, 10.5, and 10.6, whereas urban codes included 1.0, 1.1, 2.0, 2.1, 3.0, 4.1, 5.1, 7.1, 8.1, and 10.1.

^c^ Of those who completed surveys.

### Patients Approached for RSM

**FIG 1.**
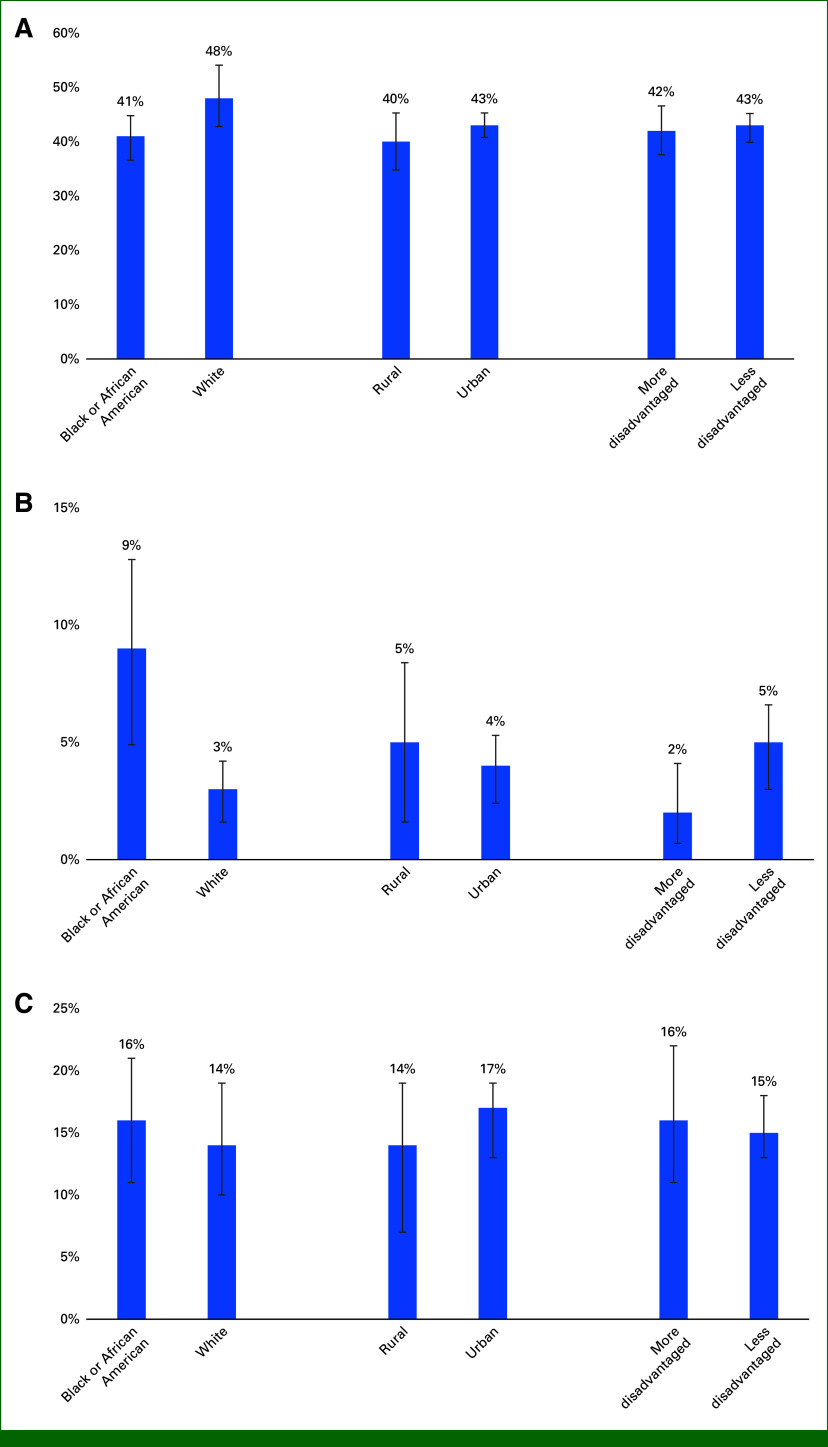
Predicted probabilities from the models evaluating the association between race, rurality, and neighborhood disadvantage and (A) being approached to participate in the RSM program, (B) declining to participate, and (C) withdrawing from RSM. All models contain race, Rural-Urban Commuting Area, Area Deprivation Index, age at approach, sex, and insurance. RSM, remote symptom monitoring.

Between May 2021 and May 2023, 883 patients receiving cancer treatment were approached for RSM because of both planned scaling with sequential rollout by providers/clinics and staffing limitations. Approached patients were more often younger than those not approached (median age, 61 *v* 64 years; Cohen's *d* = 0.18; Table 1). In adjusted models, all patient population subgroups had similar likelihoods of being approached for RSM participation (Table 2). Predicted probabilities of being approached for RSM were also similar between patient population subgroups (range, 41%-51%; Fig 1A).

**TABLE 2.** RR and 95% CI From Four Models Evaluating Associations Between Patient Race, Rurality, and Neighborhood Disadvantage and RSM Participation Outcomes

Outcome	RR (95% CI)
Approached to participate in RSM (n = 1,998)	
Black or African American race *v* White race	0.94 (0.84 to 1.06)
Rural *v* urban residence	1.07 (0.93 to 1.23)
More *v* less neighborhood disadvantage	0.99 (0.87 to 1.12)
Declined RSM participation (n = 859)^a^	
Black or African American race *v* White race	**3.09 (1.73 to 5.53)**
Rural *v* urban residence	0.77 (0.39 to 1.54)
More *v* less neighborhood disadvantage	0.49 (0.24 to 1.02)
Adherence to RSM (n = 816)	
Black or African American race *v* White race	1.07 (0.92 to 1.26)
Rural *v* urban residence	1.08 (0.89 to 1.33)
More *v* less neighborhood disadvantage	**0.81 (0.68 to 0.97)**
Withdrawing from RSM (n = 827)	
Black or African American race *v* White race	1.04 (0.70 to 1.56)
Rural *v* urban residence	1.24 (0.76 to 2.02)
More *v* less neighborhood disadvantage	1.07 (0.71 to 1.61)

NOTE. Bold values represent statistically significant differences at a .05 alpha level. All models contain race, Rural-Urban Commuting Area (rural or urban residence), Area Deprivation Index (more or less neighborhood disadvantage), age at approach, sex, insurance status, and cancer center.

Abbreviations: RR, risk ratios; RSM, remote symptom monitoring.

^a^ Other race individuals were removed because of separation of datapoints.

### Patients Declining Enrollment into RSM

Of 883 patients approached, only 56 (6%) declined to participate (Table 1). Differential enrollment between patient population groups was observed. Compared with those enrolled, patients who declined RSM enrollment were more often older (median age, 69 *v* 60 years; *d* = 0.47), Black (41% *v* 27%; Cramer's *V* = 0.08), and Medicare-enrolled (57% *v* 37%; *V* = 0.11). Adjusted models showed that Black patients were three times more likely to decline RSM participation than White patients (RR, 3.09 [95% CI, 1.73 to 5.53]; Table 2). Patients living in rural neighborhoods had 23% lower likelihood of declining RSM participation than those living in urban neighborhoods (RR, 0.77 [95% CI, 0.39 to 1.54]) although this comparison was not statistically significant. However, patients living in more disadvantaged neighborhoods had 51% lower likelihood of declining enrollment when compared with those living in less disadvantaged neighborhoods (RR, 0.49 [95% CI, 0.24 to 1.02]). Predicted probabilities ranged from 2% to 9% (Fig 1B).

### Patients' Adherence With RSM

**FIG 2.**
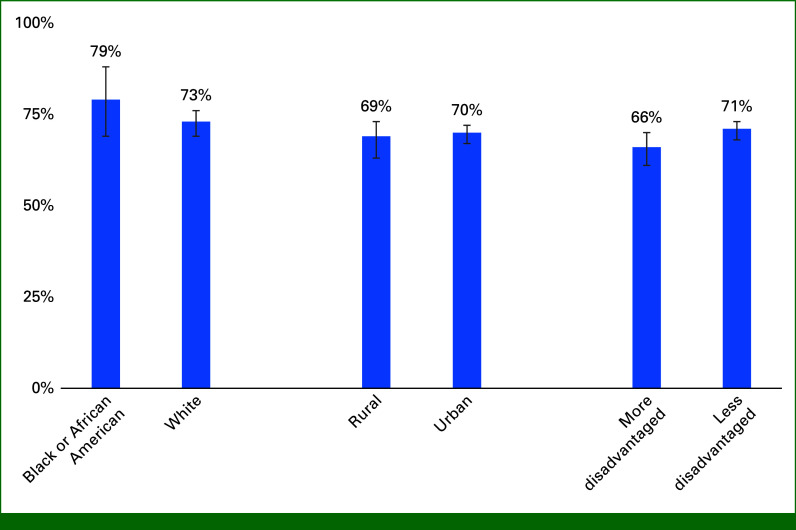
Model-estimated mean adherence percentage for RSM surveys by race, rurality, and neighborhood disadvantage among ever enrolled patients. All models contain race, Rural-Urban Commuting Area, Area Deprivation Index, age at approach, sex, and insurance. RSM, remote symptom monitoring.

Of 827 enrolled patients, 99% (n = 816) had adherence data available. Median RSM adherence was 80% (IQR, 44%-100%). In the adjusted model, similar likelihood of RSM adherence was found comparing patients who were Black versus White (RR, 1.07 [95% CI, 0.92 to 1.26]) and those residing in a rural versus urban neighborhood (RR, 1.08 [95% CI, 0.89 to 1.33]; Table 2). Conversely, patients living in more disadvantaged neighborhoods had 19% lower likelihood of RSM adherence compared with those in less disadvantaged neighborhoods (RR, 0.81 [95% CI, 0.68 to 0.97]). Model-adjusted mean adherence was 66% (95% CI, 61% to 70%) for patients living in more disadvantaged neighborhoods and 71% (95% CI, 68% to 73%) for those living in less disadvantaged neighborhoods (Fig 2).

### Patients Withdrawing From RSM

Of 827 enrolled patients, 16% (n = 133) withdrew. In adjusted models, Black versus White, rural versus urban, and more versus less disadvantaged patients had similar likelihoods of withdrawing from the study (Table 2). Model-estimated probabilities of withdrawing from RSM were similar across patient population groups (range, 14%-17%; Fig 1C).

### Complete Case and Interaction Analysis

**FIG A1.**
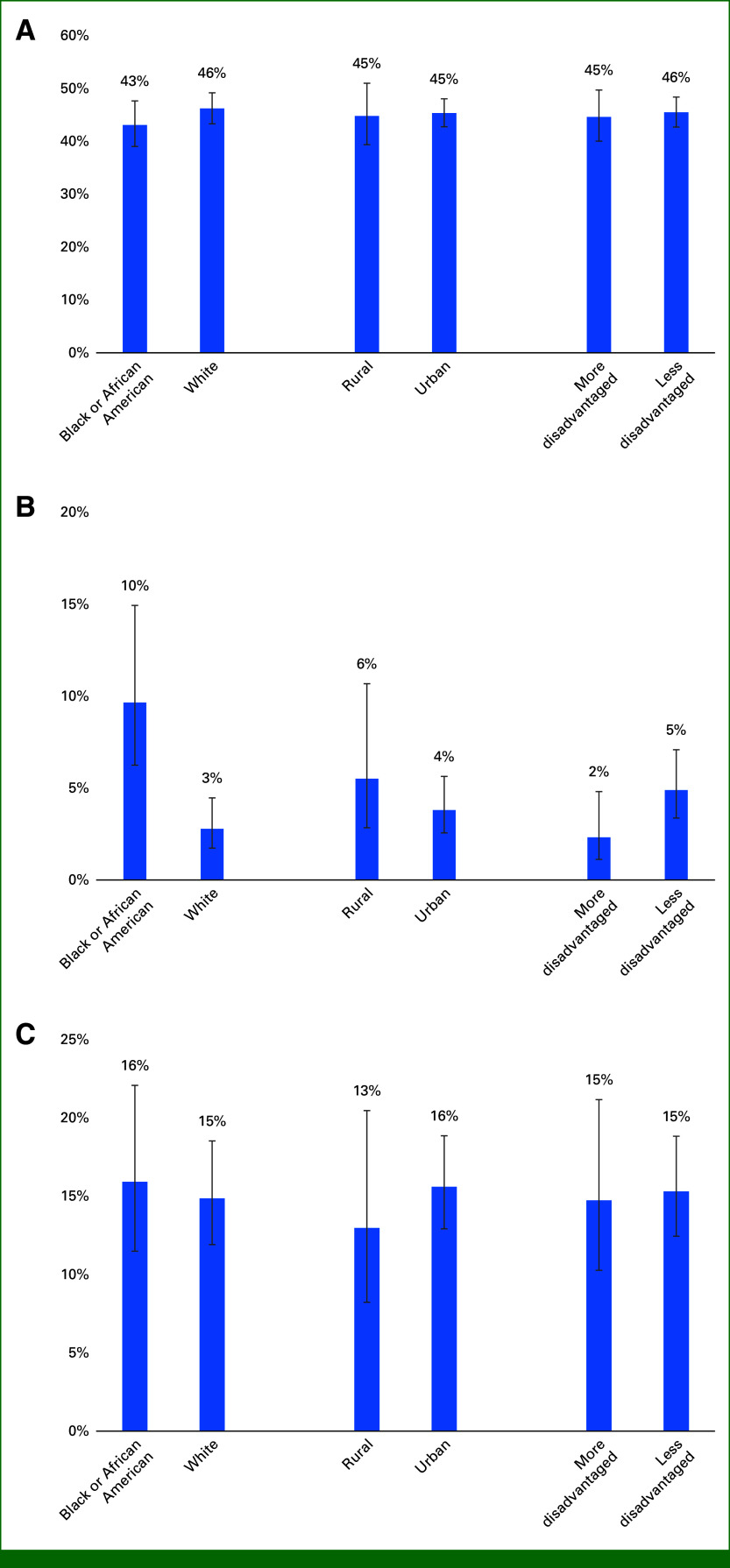
Complete case: predicted probabilities from the models evaluating the association between race, rurality, and neighborhood disadvantage and (A) being approached to participate in RSM, (B) declining to participate, (C) withdrawing from the study. All models contain race, Rural-Urban Commuting Area, Area Deprivation Index, age at approach, sex, and insurance. RSM, remote symptom monitoring.

**FIG A2.**
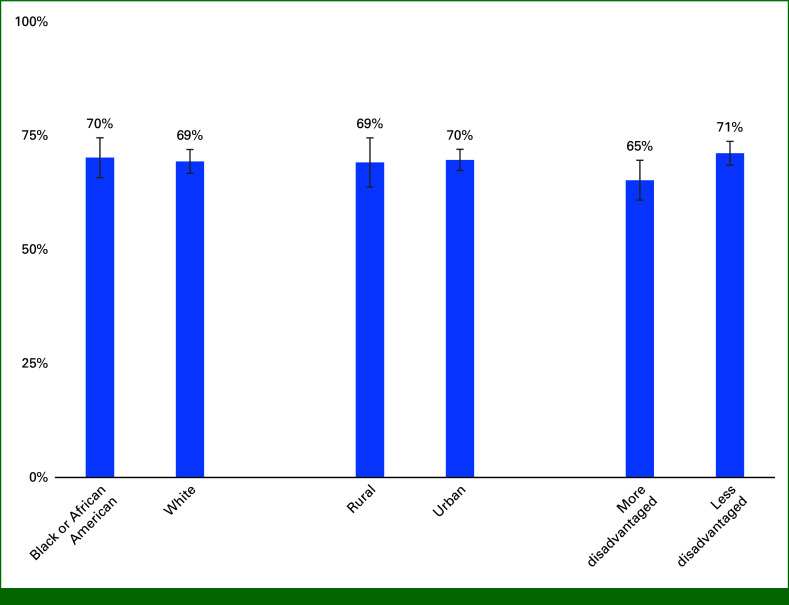
Complete case: model-estimated mean adherence percentage of RSM surveys by race, rurality, and neighborhood disadvantage among ever enrolled patients. All models contain race, Rural-Urban Commuting Area, Area Deprivation Index, age at approach, sex, and insurance. RSM, remote symptom monitoring.

Complete case demographic and clinical characteristics were similar (Appendix Table A2). Complete case model results were similar for each model (Appendix Table A3 and Figs A1 and A2). When considering the interaction between race and ADI, results were similar (Appendix Table A4).

## DISCUSSION

This study demonstrates that the majority (94%) of patients were willing to participate in RSM when approached and completion of surveys was high (80% survey completion), highlighting a general willingness of patients to engage in ePROs when offered as part of standard of care. Participation was high regardless of patient population although differential patterns of engagement were observed in RSM in a diverse sample of patients with cancer. This is problematic in the setting of an intervention with clear patient benefits, where differential engagement has the potential to exacerbate disparities in health outcomes.

It was noteworthy that patients approached did not differ by race, rurality, or socioeconomic status in this initial implementation period of RSM. This contrasts with previous clinical trials, where Black patients and those in rural neighborhoods participated less than White patients living in urban neighborhoods.^27-29^ While this juxtaposition may be secondary to the standard-of-care nature of the intervention, previous screening interventions and treatments have not always been delivered equally, as noted above.^11,12^ The success of equal engagement from the clinical teams may be related to the use of lay navigators as the primary contact point for the intervention. Previous research demonstrated that a lay navigation program focused on reducing disparities was able to increase participation of Black patients in clinical trials from 9% to 16%. In addition, Black patients receiving navigation services were more likely to complete trials when compared with those who did not receive navigation (75% *v* 38%).^30^

The use of population-based screening methods (eg, reviewing clinic lists, abstractions from the EMR and billing records) to systematically identify eligible patients for RSM enrollment rather than relying on oncologist referral also likely supports equitable enrollment. While data on oncologist referral to RSM are lacking, referral of diverse populations to clinical trials was previously shown to be suboptimal. In one cancer center, clinical trials were offered to 21% of Black women, significantly less than White women (42%).^31^ Our findings highlight the opportunity for health systems to incorporate system-based approaches to ensure that patients have equal access to RSM and other promising health care delivery interventions. However, the patterns of engagement (enrollment, survey completion, withdrawal) beyond the initial approach differed across patient populations. While Black patients were equally as likely as White patients to be approached to participate and be adherent in the program when enrolled and the majority of patients did enroll, Black patients had three times the likelihood of declining enrollment. Although we did not explicitly capture the reasons for refusal, qualitative work is ongoing to understand barriers to participation and guide further strategies for engagement and narrow engagement disparities. For the small proportion of patients declining enrollment, we anticipate that differences in engagement with technology across racial groups may be important for RSM given the reliance on e-mail and text-message delivery. In the 2019 national census, 19% of Black Americans had no access to the internet, compared with 10% of White Americans. This gap was further widened for Black Americans living below the federal poverty line (32%) or in nonmetropolitan areas (32%). Differences in access likely contributed to the finding by Irfan et al^32^ that Black patients with cancer in Alabama were less likely than White patients with cancer to report comfort with technology. Furthermore, Black Americans express concerns over data privacy. Compared with White Americans, Black Americans are more likely to believe that the government is tracking all or most of what they do online or on their cellphone (47% *v* 19%).^32^ Given these differences, interventions to address digital literacy and digital health literacy are needed to ensure equitable access to novel technology-based health interventions such as RSM.

By contrast, patients living in more disadvantaged neighborhoods were more likely to enroll, but less likely to be adherent with RSM, which may represent a passive decline. This finding aligns with previous research showing that patients with lower income are less likely to complete health care–related surveys.^33^ This may be secondary to power dynamics between the health care team and patients. Patients may be subject to social desirability bias in which agreeing to participate is perceived to be the expected and approportionate response to a participation request; they may not be comfortable expressing that they are uninterested, unwilling, unable, or may be concerned that refusal to participate may negatively affect their relationship with the health care team. Lower adherence rates may also reflect the reality of competing demands on time and resources for more disadvantaged patients with cancer, who may worry about housing stability, food insecurity, and employment.^34^ Thus, it is important to consider that barriers may differ for specific populations and further work is needed to both elicit these barriers and develop appropriate interventions.

While differences were observed by race and socioeconomic status, there was no significant difference in engagement observed for patients residing in rural versus urban areas. Although they may share some of the challenges with other marginalized populations, patients living in rural neighborhoods may be more inclined to participate because of a desire for accessible interventions that minimize travel burden and the need to get care outside their cancer center. In our previous study, patients who traveled for >1 hour had a 10% greater patient cost responsibility and were more likely to seek care locally than those whose travel time was <30 minutes.^35^

This study has several limitations. First, this analysis does not include qualitative assessment of reasons why individual patients declined participation, which is planned in future analysis. In addition, generalizability may be limited because of the unique cultural setting of these two sites in the Southeastern United States where social determinants of health and cultural characteristics may influence willingness to agree to interventions recommended by the clinical team.^36^ This analysis focused exclusively on Black versus White race and did not assess other races or ethnicities because of low prevalence of these subgroups in the catchment area. Furthermore, this analysis represents early implementation in the initial 2 years when not all patients are approached to participate because of planned slow scale-up with goals of 30% in year 1 and 40% in year 2. Despite the use of adjusted models in this analysis, the inherent intersectionality of these populations means that our findings may not fully represent individual patient experience and future interventions may need to address both willingness to participate up front and throughout the intervention.

In conclusion, Black patients and individuals living in more disadvantaged neighborhoods are at risk for lower engagement in RSM. Further work is needed to understand specific barriers to engagement and to select implementation strategies to overcome obstacles that contribute to disparate RSM outcomes.

## Data Availability

Data will be made available upon request and appropriate permissions.

## APPENDIX

**TABLE A1.** Demographics and Clinical Characteristics of Patients Eligible for (n = 1,998), Approached for (n = 883), Not Approached for (n = 1,157), Declined to Enrollment in (n = 56), and Enrolled Into (n = 827) RSM

Characteristic	Eligible (n = 1,998)	Approached (n = 883)	Not Approached (n = 1,115)	Cramer's *V* for Approached *v* Not Approached	Declined (n = 56)	Enrolled (n = 827)	Cramer's *V* for Declined *v* Enrolled	Withdrawn (n = 133)
Age at approach or eligibility state (for those not approached), years, median (IQR)	62 (52-70) n = 1,955^a^	61 (51-69)	64 (53-72) n = 1,072^a^	Cohen's *d* = 0.18	69 (58-72)	60 (51-69)	Cohen's *d* = 0.47	60 (51-68)
Approached status, No. (%)				1.00			NA	
Approached	883 (44)	883 (100)	0		56 (100)	827 (100)		133 (100)
Not approached	1,115 (56)	0	1,115 (100)		0	0		0
Declined status,^a^ No. (%)				1.00			1.00	
Declined	56 (3)	56 (6)	0		56 (100)	0		0
Enrolled	827 (42)	827 (94)	0		0	827 (100)		133 (100)
Enrollment status,^a^ No. (%)				1.00			1.00	
Enrolled	694 (35)	694 (79)	0		0	694 (84)		0
Withdrawn	133 (7)	133 (15)	0		0	133 (16)		133 (100)
Race, No. (%)				0.06			0.08	
Black or African American	527 (26)	250 (28)	277 (25)		23 (41)	227 (27)		39 (29)
Other	75 (4)	24 (3)	51 (5)		0	24 (3)		5 (4)
White	1,244 (62)	586 (66)	658 (59)		31 (55)	555 (67)		87 (65)
Unknown	152 (8)	23 (3)	129 (12)		2 (4)	21 (3)		2 (2)
Rural-Urban Commuting Area,^b^ No. (%)				0.03			0.02	
Rural	318 (16)	132 (15)	186 (17)		10 (18)	122 (15)		16 (12)
Urban	1,541 (77)	704 (80)	837 (75)		43 (77)	661 (80)		107 (80)
Unknown	139 (7)	47 (5)	92 (8)		3 (5)	44 (5)		10 (8)
Area Deprivation Index, No. (%)				0.01			0.04	
Less disadvantaged	1,365 (68)	617 (70)	748 (67)		42 (75)	575 (70)		88 (66)
More disadvantaged	477 (24)	212 (24)	265 (24)		10 (18)	202 (24)		34 (26)
Unknown	156 (8)	54 (6)	102 (9)		4 (7)	50 (6)		11 (8)
Sex, No. (%)				0.15			0.11	
Female	1,172 (59)	603 (68)	569 (51)		27 (48)	576 (70)		99 (74)
Male	783 (39)	280 (32)	503 (45)		29 (52)	251 (30)		34 (26)
Unknown	43 (2)	0	43 (4)		0	0		0
Cancer type, No. (%)				0.30			0.21	
Breast	478 (24)	290 (33)	188 (17)		9 (16)	281 (34)		58 (44)
GI	361 (18)	158 (18)	203 (18)		24 (43)	134 (16)		22 (17)
Genitourinary	125 (6)	41 (5)	84 (8)		3 (5)	38 (5)		4 (3)
Gynecologic	190 (10)	121 (14)	69 (6)		2 (4)	119 (14)		12 (9)
Head and neck	73 (4)	24 (3)	49 (4)		2 (4)	22 (3)		5 (4)
Hematologic	466 (23)	120 (14)	346 (31)		13 (23)	107 (13)		13 (10)
Lung	137 (7)	79 (9)	58 (5)		2 (4)	77 (9)		14 (11)
Melanoma	73 (4)	23 (3)	50 (4)		0	23 (3)		1 (1)
Other	44 (2)	27 (3)	17 (2)		1 (2)	26 (3)		4 (3)
Unknown	51 (3)	0	51 (5)		0	0		0
Insurance status, No. (%)				0.11			0.11	
Medicaid	153 (8)	82 (9)	71 (6)		2 (4)	80 (10)		17 (13)
Medicare	781 (39)	335 (38)	446 (40)		32 (57)	303 (37)		49 (37)
None/other	251 (13)	82 (9)	169 (15)		6 (11)	76 (9)		7 (5)
Private	810 (41)	384 (43)	426 (38)		16 (29)	368 (44)		60 (45)
Unknown	3 (0)	0	3 (0)		0	0		0
Cancer center, No. (%)				0.16			0.12	
UAB	1,468 (73)	577 (65)	891 (80)		49 (88)	528 (64)		92 (69)
MCI	530 (27)	306 (35)	224 (20)		7 (13)	299 (36)		41 (31)
Compliance percentage, median (IQR)						80 (44-100) n = 816^a^	NA	83 (52-100)
Compliance percentage, dichotomized, No. (%)							NA	
75% or greater						436 (53)		79 (59)
Below 75%						380 (47)		54 (41)

NOTE. 2.8% without information on any variables of interest.

Abbreviations: MCI, Mitchell Cancer Institute; NA, not available; RSM, remote symptom monitoring; UAB, University of Alabama at Birmingham.

^a^ Does not add up to 100% for all columns.

**TABLE A2.** Complete Case Analysis: Demographics and Clinical Characteristics of Patients Eligible for (n = 1,669), Approached for (n = 784), Not Approached for (n = 885), Declined to Enrollment in (n = 50), and Enrolled Into (n = 734) RSM

Characteristic	Eligible (n = 1,669)	Approached (n = 784)	Not Approached (n = 885)	Declined (n = 50)	Enrolled (n = 734)	Withdrawn (n = 115)
Age at approach or eligibility stare (for those not approached), years, median (IQR)	62 (52-70)	61 (51-69)	64 (53-72)	68 (58-72)	60 (51-69)	58 (50-68)
Approached status, No. (%)						
Approached	784 (47)	784 (100)		50 (100)	734 (100)	115 (100)
Not approached	885 (53)		885 (100)			
Declined status,^a^ No. (%)						
Declined	50 (3)	50 (6)		50 (100)		
Enrolled	734 (44)	734 (94)			734 (100)	115 (100)
Enrollment status,^a^ No. (%)						
Enrolled	619 (37)	619 (79)			619 (84)	
Withdrawn	115 (7)	115 (15)			115 (16)	115 (100)
Race, No. (%)						
Black or African American	500 (30)	234 (30)	266 (30)	22 (44)	212 (29)	35 (30)
White	1,169 (70)	550 (70)	619 (70)	28 (56)	522 (71)	80 (70)
Rural-Urban Commuting Area, No. (%)						
Rural	284 (17)	127 (16)	157 (18)	10 (20)	117 (16)	16 (14)
Urban	1,385 (83)	657 (84)	728 (83)	40 (80)	617 (84)	99 (86)
Area Deprivation Index, No. (%)						
Less disadvantaged	1,233 (74)	582 (74)	651 (74)	40 (80)	542 (74)	85 (74)
More disadvantaged	436 (26)	202 (26)	234 (26)	10 (20)	192 (26)	30 (26)
Sex, No. (%)						
Female	1,008 (60)	537 (68)	471 (53)	26 (52)	511 (70)	84 (73)
Male	661 (40)	247 (32)	414 (47)	24 (48)	223 (30)	31 (27)
Cancer type, No. (%)						
Breast	404 (24)	253 (32)	151 (17)	8 (16)	245 (33)	50 (43)
GI	315 (19)	135 (17)	180 (20)	21 (42)	114 (16)	20 (17)
Genitourinary	107 (6)	35 (4)	72 (8)	3 (6)	32 (4)	4 (3)
Gynecologic	165 (10)	108 (14)	57 (6)	2 (4)	106 (14)	7 (6)
Head and neck	70 (4)	22 (3)	48 (5)	2 (4)	20 (3)	4 (3)
Hematologic	379 (23)	110 (14)	269 (30)	11 (22)	99 (13)	12 (10)
Lung	126 (8)	77 (10)	49 (6)	2 (4)	75 (10)	14 (12)
Melanoma	63 (4)	20 (3)	43 (5)		20 (3)	1 (1)
Other	40 (2)	24 (3)	16 (2)	1 (2)	23 (3)	3 (3)
Insurance status, No. (%)						
Medicaid	136 (8)	75 (10)	61 (7)	2 (4)	73 (10)	14 (12)
Medicare	674 (40)	297 (38)	377 (43)	29 (58)	268 (37)	41 (36)
None/other	167 (10)	73 (9)	94 (11)	5 (10)	68 (9)	6 (5)
Private	692 (41)	339 (43)	353 (40)	14 (28)	325 (44)	54 (47)
Cancer center, No. (%)						
UAB	1,252 (75)	513 (65)	739 (84)	43 (86)	470 (64)	82 (71)
MCI	417 (25)	271 (35)	146 (16)	7 (14)	264 (36)	33 (29)
Compliance percentage, median (IQR)^b^					80 (44-100)	88 (60-100)
Compliance percentage, dichotomized, No. (%)						
75% or greater					389 (54)	73 (63)
Below 75%					337 (46)	42 (37)

NOTE. 2.8% without information on any variables of interest.

Abbreviations: MCI, Mitchell Cancer Institute; RSM, remote symptom monitoring; UAB, University of Alabama at Birmingham.

^a^ Does not add up to 100% for all columns.

**TABLE A3.** Complete Case: RR and 95% CI From Four Models Evaluating Associations Between Patient Race, Rurality, and Neighborhood Disadvantage and RSM Participation Outcomes

Outcome	RR (95% CI)
Approached to participate in RSM (n = 1,669)	
Black or African American race *v* White race	0.93 (0.83 to 1.05)
Rural *v* urban residence	0.99 (0.86 to 1.14)
More *v* less neighborhood disadvantage	0.98 (0.86 to 1.11)
Female *v* male	**1.32 (1.17 to 1.48)**
Medicaid *v* private insurance	1.00 (0.85 to 1.18)
Medicare *v* private insurance	1.00 (0.87 to 1.140
None/other *v* private insurance	0.93 (0.77 to 1.12)
MCI *v* UAB	**0.64 (0.58 to 0.71)**
Age at approach (one-unit increase)	0.99 (0.98 to 1.00)
Declined RSM participation (n = 784)	
Black or African American race *v* White race	**3.48 (1.89 to 6.36)**
Rural *v* urban residence	1.45 (0.73 to 2.88)
More *v* less neighborhood disadvantage	**0.48 (0.23 to 0.99)**
Female *v* male	**0.54 (0.32 to 0.93)**
Medicaid *v* private insurance	0.91 (0.22 to 3.72)
Medicare *v* private insurance	1.39 (0.62 to 3.11)
None/other *v* private insurance	1.47 (0.57 to 3.80)
MCI *v* UAB	**0.28 (0.13 to 0.59)**
Age at approach (one-unit increase)	**1.04 (1.01 to 1.07)**
Adherence to RSM (n = 726)	
Black or African American race *v* White race	1.08 (0.92 to 1.27)
Rural *v* urban residence	0.96 (0.79 to 1.18)
More *v* less neighborhood disadvantage	**0.79 (0.66 to 0.94)**
Female *v* male	**1.51 (1.27 to 1.80)**
Medicaid *v* private insurance	**0.74 (0.56 to 0.98)**
Medicare *v* private insurance	0.89 (0.74 to 1.06)
None/other *v* private insurance	0.77 (0.59 to 1.02)
MCI *v* UAB	**1.17 (1.03 to 1.34)**
Age at approach (one-unit increase)	1.00 (0.99 to 1.01)
Withdrawing from RSM (n = 734)	
Black or African American race *v* White race	1.07 (0.71 to 1.61)
Rural *v* urban residence	0.83 (0.51 to 1.36)
More *v* less neighborhood disadvantage	0.96 (0.63 to 1.47)
Female *v* male	1.12 (0.76 to 1.66)
Medicaid *v* private insurance	1.21 (0.70 to 2.06)
Medicare *v* private insurance	0.87 (0.56 to 1.34)
None/other *v* private insurance	0.55 (0.24 to 1.24)
MCI *v* UAB	0.70 (0.48 to 1.02)
Age at approach (one-unit increase)	1.01 (0.99 to 1.02)

NOTE. Bold values represent statistically significant differences at a .05 alpha level. All models contain race, Rural-Urban Commuting Area (rural or urban residence), Area Deprivation Index (more or less neighborhood disadvantage), age at approach, sex, insurance status, and cancer center.

Abbreviations: RR, risk ratios; RSM, remote symptom monitoring; MCI, Mitchell Cancer Institute; UAB, University of Alabama at Birmingham.

**TABLE A4.** Interaction Analysis: Odds Ratios and 95% CI From Four Models Evaluating Associations Between the Interaction of Patient Race and Neighborhood Disadvantage and RSM Participation Outcomes

Outcome	Odds Ratios (95% CI)
Approached to participate in RSM (n = 1,670)	
Less neighborhood disadvantage: Black or African American race *v* White race	0.85 (0.63 to 1.14)
More neighborhood disadvantage: Black or African American race *v* White race	0.89 (0.59 to 1.34)
Black or African American race: more *v* less neighborhood disadvantage	0.98 (0.68 to 1.42)
White race: more *v* less neighborhood disadvantage	0.94 (0.67 to 1.31)
Declined RSM participation (n = 784)^a^	
Less neighborhood disadvantage: Black or African American race *v* White race	**4.32 (2.04 to 9.12)**
More neighborhood disadvantage: Black or African American race *v* White race	3.30 (0.77 to 14.22)
Black or African American race: more *v* less neighborhood disadvantage	0.39 (0.15 to 1.05)
White race: more *v* less neighborhood disadvantage	0.51 (0.14 to 1.83)
Adherence to RSM (n = 726)	
Less neighborhood disadvantage: Black or African American race *v* White race	1.01 (0.64 to 1.61)
More neighborhood disadvantage: Black or African American race *v* White race	1.62 (0.87 to 3.01)
Black or African American race: more *v* less neighborhood disadvantage	0.76 (0.43 to 1.34)
White race: more *v* less neighborhood disadvantage	**0.48 (0.28 to 0.80)**
Withdrawing from RSM (n = 734)	
Less neighborhood disadvantage: Black or African American race *v* White race	1.16 (0.64 to 2.11)
More neighborhood disadvantage: Black or African American race *v* White race	0.96 (0.42 to 2.20)
Black or African American race: more *v* less neighborhood disadvantage	0.87 (0.42 to 1.81)
White race: more *v* less neighborhood disadvantage	1.04 (0.52 to 2.07)

NOTE. Bold values represent statistically significant differences at a .05 alpha level. All models contain race, Rural-Urban Commuting Area (rural or urban residence), Area Deprivation Index (more or less neighborhood disadvantage), age at approach, sex, insurance status, and cancer center.

Abbreviation: RSM, remote symptom monitoring.

^a^ Other race individuals were removed because of separation of datapoints.
